# Device-Based Enrichment of Knee Joint Synovial Cells to Drive MSC Chondrogenesis Without Prior Culture Expansion In Vitro: A Step Closer to 1-Stage Orthopaedic Procedures

**DOI:** 10.1177/03635465211055164

**Published:** 2021-11-15

**Authors:** Ala Altaie, Thomas G. Baboolal, Owen Wall, Hemant Pandit, Elena Jones, Dennis McGonagle

**Affiliations:** * Leeds Institute of Rheumatic and Musculoskeletal Medicine, University of Leeds, Leeds, UK; † Leeds Biomedical Research Centre, National Institute for Health Research, Leeds, UK; Investigation performed at Leeds Institute of Rheumatic and Musculoskeletal Medicine, University of Leeds, Leeds, UK

**Keywords:** synovial resident mesenchymal stem cells, platelet products, nonexpanded cells

## Abstract

**Background:**

Synovial fluid (SF) mesenchymal stem cells (MSCs) are derived from the synovial membrane and have cartilage repair potential. Their current use in clinical practice is largely exploratory. As their numbers tend to be small, therapeutic procedures using MSCs typically require culture expansion. Previous reports indicate that the stem cell–mobilizing device (STEM device) intraoperatively increases SF-MSCs.

**Purpose:**

This study evaluated the chondrogenic potential of non–culture expanded synovium-mobilized MSCs and SF-microfragments obtained after enrichment using the STEM device and ascertained if device-mediated synovial membrane manipulation facilitated ongoing MSC release.

**Study Design:**

Controlled laboratory study.

**Methods:**

Two samples of aspiration fluid were collected intraoperatively before and after STEM device utilization from patients (n = 16) undergoing diagnostic or therapeutic knee arthroscopy. Human knee synovium (n = 5) was collected during total knee replacement, and a suspended culture was performed to assess the effect of the STEM device on ongoing MSC release. Colony forming unit–fibroblastic assays were used to determine the number of MSCs. Additionally, cytometric characterization of stromal and immune cells and chondrogenesis differentiation assay were performed without culture expansion. Filtered platelet concentrates were prepared using the HemaTrate system.

**Results:**

After STEM device use, a significant increase was evident in SF-MSCs (*P* = .03) and synovial fluid–resident synovial tissue microfragments (*P* = .03). In vitro–suspended synovium released significantly more MSCs following STEM device use than nonstimulated synovium (*P* = .01). The STEM device–released total cellular fraction produced greater in vitro chondrogenesis with significantly more glycosaminoglycans (GAGs; *P* < .0001) when compared with non–STEM device synovial fluid material. Nonexpanded SF-MSCs and SF-microfragments combined with autologous filtered platelet concentrate produced significantly more GAGs than the complete chondrogenic media (*P* < .0001). The STEM device–mobilized cells contained more M2 macrophage cells and fewer M1 cells.

**Conclusion:**

Non–culture expanded SF-MSCs and SF-microfragments had the potential to undergo chondrogenesis without culture expansion, which can be augmented using the STEM device with increased MSC release from manipulated synovium for several days. Although preliminary, these findings offer proof of concept toward manipulation of the knee joint environment to facilitate endogenous repair responses.

**Clinical Relevance:**

Although numbers were small, this study highlights 3 factors relevant to 1-stage joint repair using the STEM device: increased SF-MSCs and SF-microfragments and prolonged synovial release of MSCs. Joint repair strategies involving endogenous MSCs for cartilage repair without the need for culture expansion in a 1-stage procedure may be possible.

Osteoarthritis is the most common joint disease, where severe joint degeneration occurs in association with pain and function loss. Apart from joint replacement, nonsurgical therapeutic options remain limited.^39,41,49^ For over 2 decades, there has been significant interest in joint regenerative strategies with various scaffolds, growth factors, drugs, and stem cells.^27,52,58^ Among the proposed treatments of knee osteoarthritis is the combination of multipotential stromal cells, also known as mesenchymal stem cells (MSCs), and platelet-rich plasma or platelet lysate.^13,18,38,44^ MSCs have a significant therapeutic potential for cartilage regeneration in experimental settings and, as such, have been viewed as being central to joint regeneration.^42^ The historical perception that MSCs were rare underlies culture expansion protocols before experimental use or clinical evaluation, with off-the-shelf cell products obtained from other locations, including lipoaspirates, touted as potential therapies.^25^

The human osteoarthritis joint environment has several MSC niches, including the subchondral bone, synovial membrane, joint fat, and synovial fluid (SF).^11,21,22,54^ Although readily accessible upon joint aspiration, a major limitation to the use of resident SF-MSCs is the perception that they need to be removed from the joint and culture expanded to generate clinically relevant quantities before reintroduction.^23^ Nevertheless, SF-MSCs quickly attach to the diseased cartilage in canine models, supporting their potential role in joint repair.^5^

Baboolal et al^4^ showed that mechanical agitation of the knee synovium using a purpose-made stem cell mobilizing device (the “STEM device”) during arthroscopy led to a considerable increase in MSCs, termed synovial-mobilized MSCs (Sm-MSCs). However, other factors have not been considered regarding the use of the STEM device. Suspended synovial tissue has been shown capable of extending MSC release into culture media, pointing toward a potential dynamic and sustained mechanism of MSC access to the joint cavity and cartilage surfaces.^26^ Therefore, adult synovium may serve as a potential source for “seeding” the SF and provide a source of MSCs to superficial cartilage and other damaged joint structures for repair, akin to how stem cells are thought to seed the cartilage during embryological development.^26^ The effect of the STEM device on MSC release with the potential to enrich joint cavity MSCs has not been considered. Furthermore, the STEM cell device may be capable of releasing microfragments of synovium and not just single cells. This is potentially important, as SF-resident synovial microfragments (SF-microfragments) are a novel source of SF-MSCs that could contribute to MSC-mediated joint repair without culture expansion.^10,57^

In addition to SF-MSCs, SF contains immune cells, such as monocyte/macrophages, lymphocytes, granulocytes, and natural killer cells.^17^ Macrophages present classically activated (M1) or alternatively activated (M2) phenotypes, exhibiting proinflammatory or anti-inflammatory properties, respectively.^30,37^ M1 macrophages are characterized by surface expression of the costimulatory molecule CD86, and the M2 phenotype is associated with surface expression of the mannose receptor (CD206).^14^ M1 macrophages have an antichondrogenic effect, while the M2 phenotype enhances cartilage repair.^14^

Platelet-rich plasma / platelet lysate can enhance the chondrogenic activity of SF-MSCs via growth factors that promote homeostasis of joint tissues through chondroprotective, anabolic, anti-inflammatory, and immunomodulatory effects.^3,9,35,45,55^ Autologous platelet concentrates (PCs) represent an endogenous growth factor milieu for assessing how native joint stem cells may behave in an in vitro system.

This study evaluated the chondrogenic potential of non–culture expanded Sm-MSCs and SF-microfragments obtained after enrichment using the STEM device and ascertained if device-mediated synovial membrane manipulation facilitated ongoing MSC release. We undertook these experiments under physiologically compatible, clinically relevant material using autologous PCs and linking this to the immune status of resident SF macrophages.

## Methods

### Participants and Samples

Ethical approval for this study was granted by Yorkshire and the Humber–South Yorkshire Research Ethics Committee (14/YH/0087), in compliance with the Helsinki Declaration of ethical principles for medical research involving human participants. All study participants provided informed consent. All patients underwent elective knee diagnostic or therapeutic arthroscopy (n = 16; median age, 31 years; range, 18-55 years). At the time of arthroscopy, patients were free from joint effusion, synovitis, or active inflammation.

### Retrieval of MSCs From Aspiration Fluid and Mobilization of Synovial MSCs

The first sample contained resident SF-MSCs, and the second aspirate sample contained Sm-MSCs, both of which were retrieved and collected as previously described.^4^ The aspirated fluid was centrifuged (500 rcf [relative centrifugal force] for 5 minutes), and cells were resuspended in 10 mL of Dulbecco’s modified Eagle medium (DMEM) containing 100 U/mL of penicillin and 100 mg/mL of streptomycin (all from Invitrogen); the SF cells were split according to the experimental design shown in Appendix Figure A1 (available in the online version of this article). For MSC expansion, cells were cultured in StemMACS expansion medium (Miltenyi Biotec), containing penicillin and streptomycin with twice-weekly media changes, and expanded for 3 or 4 passages. Moreover, donor-matched cultures were used in all experiments when they reached passage 3.

### Quantification of MSC Colony Number and SF-Microfragments

To determine the number of viable MSCs in each sample, a colony forming unit–fibroblastic (CFU-F) assay was used as previously described.^4,22^ After 2 weeks of culture, colonies were stained with 1% methylene blue (Sigma-Aldrich) and analyzed using ImageJ Version 2.0 (National Institutes of Health). For SF-microfragment quantification, colonies were examined using a light microscope, and colonies with a high density of cells were considered SF-microfragments.

### Preparation of Autologous and Allogenic PC

A gravity-based blood filtration system (HemaTrate; CookRegentec) was used for preparing filtered PC (fPC). According to the manufacturer’s instructions, 60 mL of blood was collected before the arthroscopy (autologous [AUT-fPC]; n = 5) or from healthy volunteers (allogenic [ALL-fPC]; n = 6), resulting in 8 mL of fPC. Two freeze-and-thaw cycles were applied to release the growth factors from platelets at −80°C for 24 hours, followed by thawing at 37°C. Quantification of platelets, white blood cells, and red blood cells in 300 µL of whole blood or 300 µL of fPC was performed using a Sysmex hematology analyzer.

### fPC as a Chondrogenic Inducer for Culture-Expanded SF-MSCs of the First Aspirate

Chondrogenic differentiation of culture-expanded SF-MSCs (first aspirate) treated with AUT-fPC or ALL-fPC as a chondrogenic inducer (n = 5) was performed as previously described.^2^ Cells were pelleted and cultured for 3 weeks in a complete chondrogenic differentiation medium (CCM; Miltenyi Biotec) as a positive control or in high-glucose DMEM medium supplemented with 50% AUT-fPC or ALL-fPC with half media change 3 times a week. Glycosaminoglycan (GAG) production was visualized on 5-µm frozen sections stained with 1% toluidine blue (Sigma). Gene expression of the chondrogenic transcripts (*SOX-9*, *ACAN*, and *COL2A1*) was quantified at weeks 0 and 3. For this, pellets were digested, and RNA was isolated (n = 3 donors for each condition) using the Total RNA Purification Kit (Norgen Bioteck). Reverse transcription was performed, followed by 14 preamplification cycles using a mixture of 48 TaqMan gene expression assays. Gene expression was measured using standard TaqMan assays (Thermo Fisher Scientific) and the 48 × 48 integrated fluidic circuit with the recommended reagents (Fluidigm), as previously described.^46^ The integrated fluidic circuit was then run on the BioMark real-time polymerase chain reaction system using a GE 48 × 48 Standard Version 1 polymerase chain reaction thermal protocol, and data were analyzed using BioMark Gene Expression Data software and normalized to the housekeeping gene hypoxanthine phosphoribosyltransferase 1.

### Chondrogenic Potential for Nonexpanded SF-MSCs With AUT-fPC

Pellet culture was used to evaluate the chondrogenesis of nonexpanded SF-MSCs (first aspirate) treated with donor-matched 50% AUT-fPC (n = 5) on day 21 of culture. This strategy was used to re-create an environment that was physiologically compatible and clinically relevant to the native human state, with CCM and high-glucose DMEM used as positive and negative controls, respectively. GAG production and toluidine blue staining of frozen sections were used for quantitative and qualitative analysis, respectively. In addition, for each donor sample, CFU-F was performed to determine the numbers of viable MSCs in each pellet.

### Chondrogenic Potential of Donor-Matched Nonexpanded SF-MSCs and Sm-MSCs

For chondrogenic potential comparisons, pellet cultures were established with nonexpanded donor-matched cells before and after STEM device use with CCM. The production of GAGs was measured with toluidine blue staining performed on day 21. Gene expression of the chondrogenic transcripts (*SOX-9*, *ACAN*, *COL2A1*, and *COMP*) and anabolic and catabolic transcripts (*MMP9*, *TIMP3*, *TGFBR2*, and *IGFBP3*) was quantified at weeks 0 and 3. CFU-F was performed alongside this to count viable MSCs in each sample, as described earlier.

### Immune Cell Characterization

Nonexpanded donor-matched cells before and after use of the STEM device (n = 6) were stained using antibodies for MSCs and macrophage markers, including CD90-Alexa700 (AbD Serotec), CD45-V450, CD14-FITIC, CD16-BV786, HLA-DR-PE, M2 marker CD206-APC, and M1 marker CD86-PECy7 (all from BD Biosciences).^14^ Isotype controls were used for each antibody with nonspecific antibody binding blockade using phosphate-buffered saline containing 10% mouse serum and 1% human IgG. For all flow cytometry analyses, gating was first established (at 1% positivity) on the isotype controls and then applied to the corresponding markers. Data were acquired and analyzed using Cytoflex S (Beckham Coulter).

### Suspended Synovial Culture

Methods developed by the Sekiya group were adapted for our purpose to determine whether synovial manipulation after the use of the STEM device was associated with prolonged MSC release into the fluid phase.^26^ Human synovium was collected during total knee replacement from 5 patients (median age, 68 y; range, 55-83 y), after which the synovium was cut into 2 pieces, approximately 1 g from each donor, and washed in phosphate-buffered saline to remove blood traces. Synovial samples were either mechanically manipulated using the STEM device for 1 minute to replicate the in vivo procedure or left with no mechanical agitation. Then, tissues were placed in individual cell strainers with 100-µm pores (Sigma), which were subsequently placed into individual wells of a 6-well plate containing StemMACS medium (the tissues were well-covered with media). After 4 days of culture, the suspended synovium was moved to a fresh cell strainer and placed into a new 6-well plate with fresh media. This process continued on days 8 and 12 until day 20. After removal of the cell strainer with tissue from each well, media were changed entirely, and the plate was cultured for 14 days with half media change 3 times a week. At the end of the culture, colonies formed from the released cells at the bottom of the plate were stained with methylene blue, and colony counting was performed (Appendix Figure A1, available online).

### Statistical Analysis

All statistical analyses were conducted in Prism Version 7 (GraphPad). The Shapiro-Wilk normality test revealed that the data were not normally distributed owing to the limited number of samples, and as a result, a Wilcoxon matched-pair test was used. For correlation analysis, a Spearman nonparametric test was used. Two-way analyses of variance were used to determine the difference among ≥3 groups with post hoc Tukey multiple-comparison tests. The confidence level for each was set at 95%. The difference between the groups was considered statistically significant only if *P* < .05.

## Results

### STEM Device Increases MSCs and SF-Microfragments

**Figure 1.**
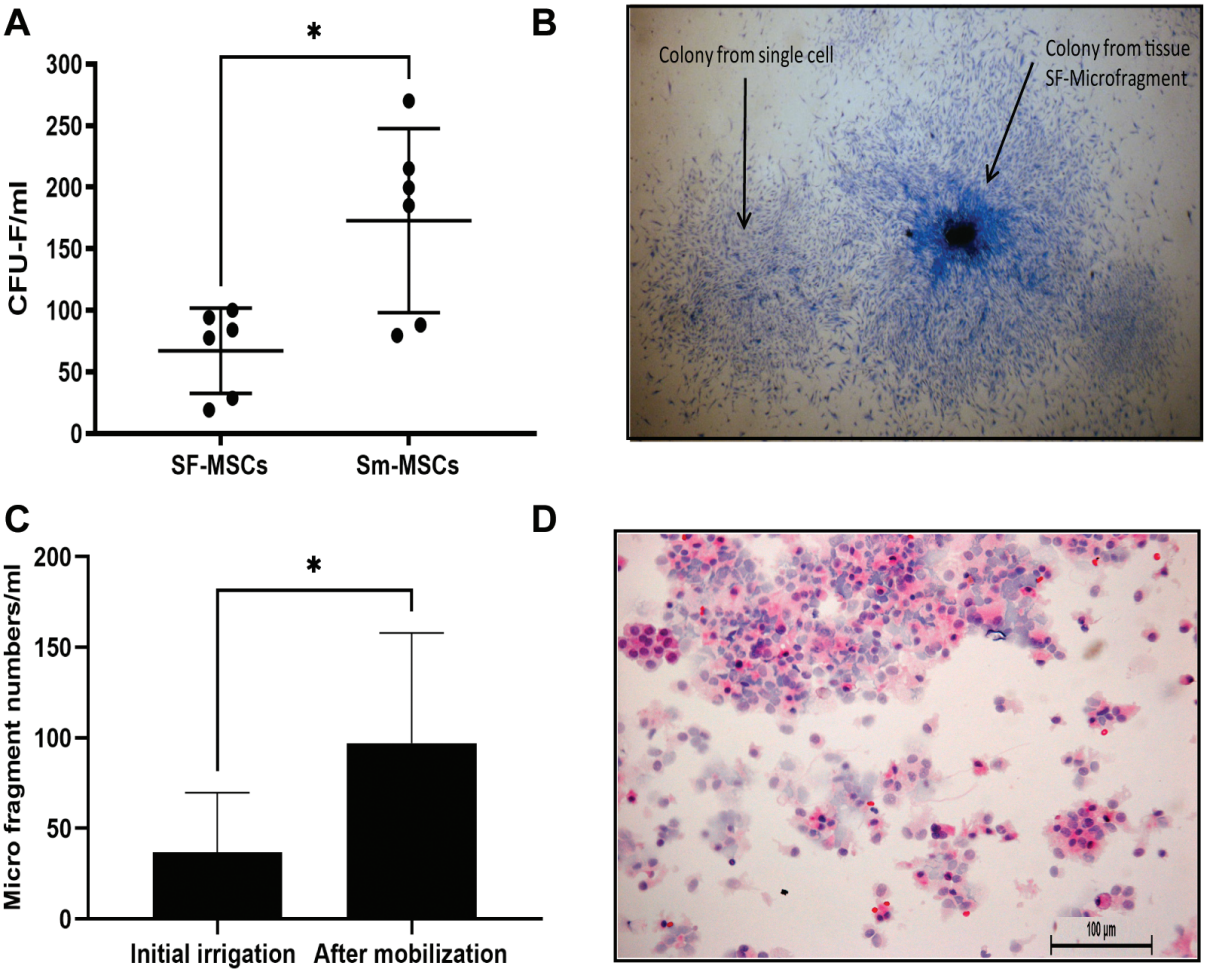
Enumeration of resistant SF-MSCs, Sm-MSCs, and SF-microfragments. (A) CFU-F numbers in aspiration and mobilized samples containing SF-MSCs and Sm-MSCs, respectively (n = 6). (B) Colonies derived from SF-MSCs and SF-microfragments. (C) SF-microfragment numbers before and after mobilization (n = 6). (D) Hematoxylin and eosin–stained SF-microfragments after mobilization. Scale bar = 100 µm. Values are presented as mean ± SD. *P < .05. CFU-F, colony-forming unit–fibroblastic; MSC, mesenchymal stem cell; SF, synovial fluid; Sm, synovial mobilized.

In agreement with the previous findings,^4^ joint cavity aspirates after the mechanical agitation of synovium using the STEM device contained significantly higher CFU-F numbers than CFU-Fs naturally occurring after joint aspiration (*P* = .03) (Figure 1A). In addition to the native resident SF-MSCs, the SF contained SF-microfragments. After STEM device mobilization, significant increases in SF-microfragments were evident (*P* = .03) (Figure 1, B and C). The hematoxylin and eosin stain of these fragments revealed large mononuclear cells with pale nuclei (Figure 1D).

### Evaluation of fPC as a Chondrogenic Inducer for Culture-Expanded SF-MSCs

**Figure 2.**
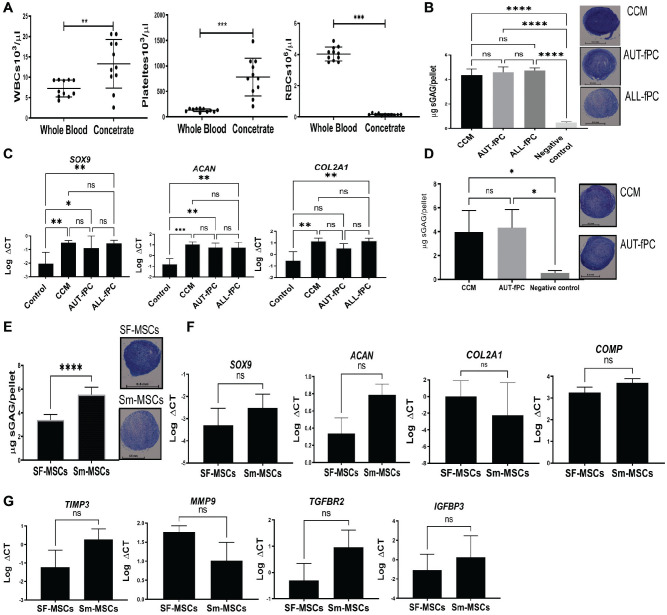
Chondrogenic potential of SF-MSCs and Sm-MSCs. (A) The number of platelets, white blood cells (WBCs), and red blood cells (RBCs) before and after filtration (n = 11). (B) Production of GAGs by culture-expanded SF-MSCs after 21 days with CCM, 50% AUT-fPC, 50% ALL-fPC, and negative control (DMEM media only) (n = 5) with representative images of toluidine blue–stained sections of SF-MSC pellets. Scale bar = 0.5 mm. (C) Expression of the chondrogenic markers in SF-MSC pellets (n = 3). (D) GAG production by SF-MSCs (from initial aspiration) after 21-day culture with CCM, AUT-fPC, and negative control (DMEM media only) (n = 5) with representative images of toluidine blue–stained sections of SF-MSC pellets. Scale bar = 0.5 mm. (E) Comparison of SF-MSC and Sm-MSC GAG production and representative images of toluidine blue–stained sections after 21-day culture with CCM (n = 6). Scale bar = 0.5 mm. (F, G) Expression of chondrogenic, anabolic, and catabolic markers (n = 3). Values are presented as mean ± SD. ns, not significant. *P < .05. **P < .01. ***P < .001. ****P < .0001. ALL, allogenic; AUT, autologous; CCM, complete chondrogenic differentiation medium; ΔCT, the difference between gene Ct and house keeping gene CT; DMEM, Dulbecco’s modified Eagle medium; fPC, filtered platelet concentrate; GAG, glycosaminoglycan; MSC, mesenchymal stem cell; SF, synovial fluid; Sm, synovial mobilized.

To assess the potential of fPCs in chondrogenesis, we first evaluated the quality of the fPCs processed by HemaTrate. The device significantly concentrated platelets (6-fold; *P* = .001) and white blood cells (1.8-fold; *P* = .006) and reduced red blood cells (26-fold; *P* = .001) (Figure 2A). The potential effect of AUT-fPC or ALL-fPC on SF-MSC chondrogenic differentiation was evaluated on culture-expanded SF-MSCs and compared with CCM (positive control) and high-glucose DMEM with no inducers (negative control).^2^ There was a significant GAG increase in pellets induced by AUT-fPC, ALL-fPC, and CCM as compared with the negative control (*P* < .0001) (Figure 2B). Comparable GAG production was evident with AUT-fPC, ALL-fPC, and CCM with comparable toluidine blue staining. This was confirmed by the gene expression of transcription factor *SOX9* and chondrogenic markers *ACAN* and *COL2A1*. Although *SOX9* and *ACAN* were significantly increased with CCM (*P* = .0043; *P* = .0006), AUT-fPC (*P* = .04 and *P* = .0033), and ALL-fPC (*P* = .005 and *P* = .0035) as compared with the negative control, a significant increase in *COL2A1* was observed with CCM (*P* = .0016) and ALL-fPC (*P* = .014) but narrowly failed significance for AUT-fPC (*P* = .06) (Figure 2C).

### Nonexpanded SF-MSC and SF-Microfragment Chondrogenic Differentiation From the First Aspirate With AUT-fPC Stimulation

Initial experiments were performed with SF-MSCs and SF-microfragments present in the first fluid aspirate from arthroscopy without culture expansion. After 21-day pellet culture, significant induction of GAGs was observed with CCM and AUT-fPC as compared with the negative control (*P* = .026 and *P* = .011, respectively) with positive toluidine blue staining of representative pellets (Figure 2D). Next, we explored the effect of increased MSC release on chondrogenesis without culture expansion. Given the limitation of fPC sample volume, only CCM was used. Accordingly, when the GAG content of SF-MSCs and the STEM device procured Sm-MSC samples induced with CCM, the GAG production was significantly higher with Sm-MSC samples as compared with SF-MSC samples (*P* < .0001) with representative stained pellets (Figure 2E). The higher levels of GAG production from Sm-MSCs positively correlated with CFU-F numbers (*r* = 0.8741; *P* < .0001) (Appendix Figure A2, available online).

To investigate chondrogenesis further, the chondrogenic gene transcripts of SF-MSCs and Sm-MSCs were determined 21 days after chondrogenic differentiation in CCM media. An increasing trend was observed with Sm-MSCs for chondrogenic markers *SOX9* (3.8-fold; *P* = .25), *ACAN* (2.3-fold; *P* = .25), *COMP* (2.6-fold; *P* = .25), and *COL2A1* (19-fold; *P* = .25) (Figure 2F) as compared with SF-MSCs, as well as notable increases in the expression of *SFRP4* (584-fold) and *GDF5* (3.5-fold), which are both highly specific for synovial-origin MSCs^4,24,48^ (Appendix Figure A3, available online). *PPARG*, which has a vital role in adipogenesis,^20^ presented 3.5-fold lower expression, and no change of the osteogenic transcription factor *Runx2* expression was found with Sm-MSCs as compared with SF-MSCs.

Additionally, the nonexpanded SF-MSC and Sm-MSC potential for regenerative therapy was examined when anabolic and anticatabolic gene expression was evaluated after 21-day chondrogenic differentiation in CCM media. Interestingly, the expression of genes involved in cartilage catabolism and extracellular matrix turnover (eg, matrix metalloproteinase 9 [*MMP9*]) was lower in Sm-MSCs than in SF-MSCs. Furthermore, overexpressed genes (>2-fold) in Sm-MSCs as compared with SF-MSCs were as follows: the tissue inhibitor of metalloproteinases 3 (*TIMP*-3), the transforming growth factor receptor 2 (*TGFBR2*), and the insulin-like growth factor binding protein 3 (*IGFBP3*), a cartilage-anabolic cytokine,^50^ by 12.4-, 18.9-, and 76.7-fold, respectively (Figure 2G).

### Cell Composition of First Aspiration and Mobilized Joint Aspirates

**Figure 3.**
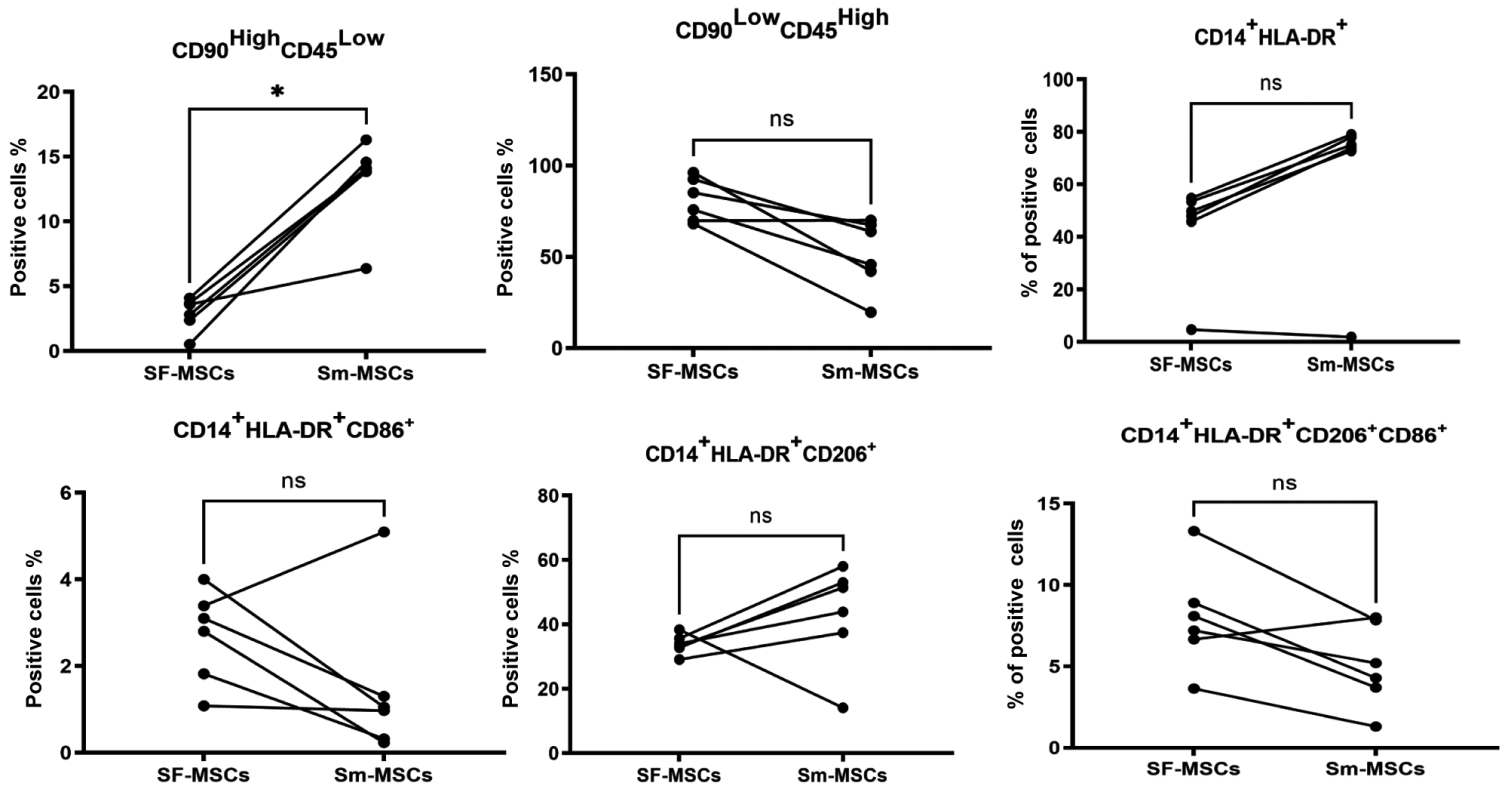
Surface marker expression of initial aspiration and mobilized joint aspirates: a comparison of relative frequencies of CD90^High^CD45^Low^ and macrophage subpopulations in nonexpanded initial aspiration and mobilized joint aspirates (n = 6). ns, not significant. *P < .05. HLA-DR, human leukocyte antigen-DR; MSC, mesenchymal stem cell; SF, synovial fluid; Sm, synovial mobilized.

Synovial membrane contains synovial macrophages and fibroblasts. It was therefore essential to evaluate cell types before and after STEM device use, with a focus on M1 macrophages (CD45^+^CD14^+^HLA-DR^+^CD86^+^) and M2 macrophages (CD45^+^CD14^+^HLA-DR^+^CD206^+^) as well as stromal lineage cells (CD90^High^CD45^Low^). The gating strategy is shown in Appendix Figure A4 (available online). There was a significant increase in the CD90^High^CD45^Low^ stromal fraction^6^ after device use (*P* = .03) (Figure 3). In 5 of 6 samples, mobilized cells showed an increase in the activated macrophages with an increase in M2 and a decrease in M1. Furthermore, there was a positive correlation between the percentage of CD90^High^CD45^Low^ cells and CFU-Fs (*r* = 0.629; *P* = .0323) and GAG production (*r* = 0.7; *P* = .007), confirming that the higher GAG activity might be due to the increase in the stromal cells/MSCs as previously indicated by CFU-F. Simultaneously, GAG production correlated positively with M2 macrophages (*r* = 0.65; *P* = .023) and negatively with M1 macrophages (*r* = −0.6; *P* = .039), the latter of which was previously reported to suppress chondrogenesis.^14^

### STEM Device Use Facilitates Sustained MSC Release

**Figure 4.**
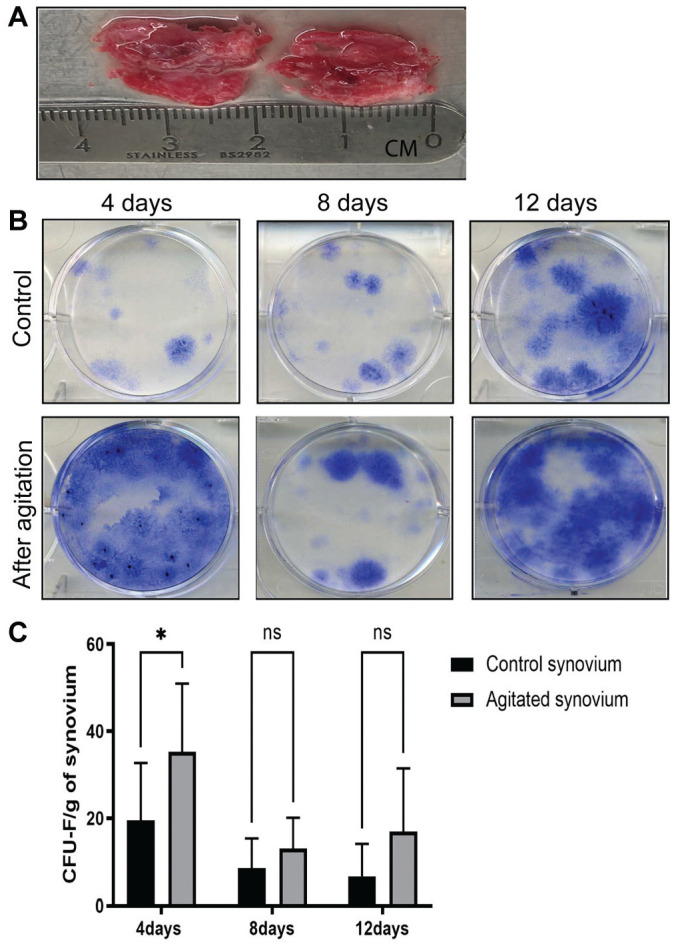
Effect of agitation on Sm-MSC release from the synovium. (A) Synovium from total knee replacement was divided into 2 parts with identical weight. (B) Methylene blue staining of colonies produced from released cells in suspended cultures. (C) Comparison of CFU-F numbers for suspended cultures for 4, 8, and 12 days of culture (n = 5). Values are presented as mean ± SD. *P < .05. CFU-F, colony forming unit–fibroblastic; ns, not significant.

As shown here, the STEM device increased the number of synovial stromal cells and M2 macrophages, both of which could contribute to higher chondrogenic potentials. However, whether synovial manipulation using the STEM device would increase the number of Sm-MSCs released into the joint cavity long term is unknown. Synovium from total knee replacements was divided into 2 parts with identical weight, with 1 piece subjected to device use and both suspended in culture, as described previously (Figure 4A).^26^ After 2 days of suspended culture, the synovium was moved to fresh media for another 2, 4, and 8 days in culture (4-, 8-, and 12-day time points). At the end of each time point, colonies formed at the bottom of the plate were quantified (Figure 4B). After the first 4 days in suspended culture, there was a significant difference in CFU-Fs released from manipulated synovium as compared with control synovium (*P* = .01) (Figure 4C). The same pattern was observed at days 8 and 12; however, no difference in CFU-Fs was seen, likely reflecting spontaneous synovial disaggregation in culture.

## Discussion

The current therapeutic strategies for knee osteoarthritis involve short-term pain relief with joint replacement for more advanced disease progression. There is an ongoing interest in regenerative medicine approaches. However, new strategies are required to limit operation time, cost, and tissue manipulation, such as abolishing the need for exogenous cell culture and fostering of 1-stage surgical procedures centered exclusively on the knee joint environment without taking tissue from exogenous sites such as the marrow or fat tissues. For the first time, this study shows that nonexpanded SF-MSCs and SF-microfragments collected during arthroscopy using a STEM device had enhanced chondrogenesis in vitro using autologous PC without in vitro expansion.^28^ This capability may be related to the SF-microfragments and SF-MSCs present in SF in greater numbers after STEM device release.

The discovery of joint-resident MSCs provides the potential of new paths for cartilage repair.^33^ Several studies have documented an increase in the SF-MSCs in patients with osteoarthritis and cartilage injury,^21,22,48^ with the same observation reported after ligamentous and meniscal damage.^32,36^ Experimental model systems have also shown that synovial MSCs may act as major contributors to cartilage repair.^43^ Thus, there are promising results in using SF-MSCs clinically. However, these studies required in vitro expansion.^1,47^ A recent study showed an increase in SF-MSCs after an arthroscopic mechanical mobilization procedure using a novel single-stage technique.^4^ The present data demonstrated that Sm-MSCs and SF-microfragments originating from the synovial membrane collected after mechanical mobilization had significantly higher GAG production than SF-MSCs, with higher expression of chondrogenic genes *SOX-9*, *COL2A1*, and *ACAN*.^15,19^ The higher GAG activity for Sm-MSCs might be due to increased CD90-positive cells, as the GAG level positively correlates with CFU-F and CD90, as previously related to enhanced chondrogenic potential.^40^

The STEM device successfully increased the number of SF-MSCs.^4^ However, the synovial membrane also contains synovial macrophages and fibroblasts; therefore, it was essential to evaluate the in vitro model of M1 and M2 synovial macrophage release to understand potential repair mechanisms relevant for cartilage repair strategies. The presence of M1 and M2 macrophages in the synovium of patients with osteoarthritis has been reported.^51^ Nevertheless, the M2 phenotype was present in the synovial lining of patients with osteoarthritis more so than M1.^17,51^ This study documents the presence of M1 and M2 populations in the joint aspirates before and after use of the STEM device, with the increased M2 following mechanical mobilization, which correlates with GAG production and chondrogenic gene expression. Simultaneously, fewer M1 macrophages had an inhibitory effect on the chondrogenic differentiation of MSCs.^14^

Finally, a suspension synovial culture model^26^ was used to show the effect of mechanical mobilization. The number of the released MSCs was higher after STEM device use of excised osteoarthritis synovium than control synovium up to day 4. These results suggest that the use of the STEM device may not stop on the day of the procedure and instead may continue releasing more MSCs into the joint cavity. The finding supports the idea that joint and synovial manipulation may augment in vivo release of native MSCs toward repair and thus needs further evaluation. The injured synovial membrane is likely capable of repair after using the STEM device since the synovium is an organ that undergoes hyperplasia in osteoarthritis and primary inflammatory arthritis. It is known to have considerable powers of regeneration, and after synovectomy, where it is severely hyperplastic, it can undergo hyperplasia once again.^31^

In the present study, we used autologous PC for proof-of-concept in vitro studies, but it is unclear if this would enhance natural regenerative joint capability in the native osteoarthritis knee joint. PCs contain growth factors that promote articular chondrocyte proliferation and GAG synthesis.^7^ Currently, PCs are prepared by different methods that include using centrifugation and gravity filtration systems.^16^ This study showed that fPC based on a gravity filtration system was significantly enriched in platelets and leukocytes and depleted in red blood cells. The PAW classification system is based on the following 3 components: (1) the absolute number of Platelets, (2) the manner in which platelet Activation occurs, and (3) the presence or absence of White cells.^12^ According to this system, fPC is classified as P3-A (platelets “high,” leukocytes “enriched above baseline”), which is superior to PCs prepared using a centrifugation system, which is classified as P2-A (platelets “moderately enriched,” leukocytes “enriched above baseline”).^34^

The platelet enrichment of fPC significantly correlated with SF-MSC proliferation. Additionally, fPC as a chondrogenic inducer significantly enhanced GAG production of SF-MSCs and Sm-MSCs. The results support previous data demonstrating that platelets contain growth factors capable of stimulating chondrocyte and chondrogenic MSC proliferation, promoting chondrocyte cartilaginous matrix secretion, and diminishing the catabolic effects of proinflammatory cytokines.^8,29,53,56^ Whether introducing fPC into the joint would augment spontaneous joint repair in different settings, such as knee joint distraction or high tibial osteotomy surgery, remains to be determined. However, we used fPC in the present study to try to create a surrogate environment ex vivo to show the differentiation of Sm-MSCs into cartilage without expansion protocols.

This study’s limitations include the small volume size of AUT-fPC that precluded chondrogenic comparison for SF-MSCs and Sm-MSCs in AUT-fPC, which was instead performed in CCM. Another important limitation was the small sample size; nevertheless, this clearly showed proof of concept of cartilage formation without MSC expansion, especially after MSC mobilization from the synovium. Yet, the potential of AUT-fPC to induce chondrogenesis was investigated using nonexpanded cells. Next, there were a limited number of donors for the gene expression data. The study’s primary purpose was to determine the potential of synovial MSCs to undergo chondrogenesis with no expansion, which can be applied in 1 stage during a range of routine arthroscopies. In this study, the MSCs were removed from the joint for laboratory analysis, so it cannot be assumed that such cells would differentiate into chondrocytes in vivo.

In conclusion, this work shows that it is possible to undertake 1-stage joint manipulation using simple devices to augment endogenous MSCs and growth factors to increase chondrogenesis from joint cavity–derived cellular and tissue SF-microfragments without cell expansion. The work supports the idea that MSCs in the fluid are likely to be released after the use of the STEM device over a longer time frame, potentially leading to a more significant number of MSCs being able to interact with injured cartilage. With the changes taking place in the joint cavity, namely the removal of antiadhesive hyaluronan, this may favor MSC adhesion to cartilage.^5^ Non–culture expanded SF-MSCs and SF-microfragments had the potential to undergo chondrogenesis without culture expansion, which can be augmented using the STEM device with increased MSC release from manipulated synovium for several days. Although preliminary, these findings offer proof of concept toward manipulation of the knee joint environment to facilitate endogenous repair responses.

## Supplemental Material

sj-pdf-1-ajs-10.1177_03635465211055164 – Supplemental material for Device-Based Enrichment of Knee Joint Synovial Cells to Drive MSC Chondrogenesis Without Prior Culture Expansion In Vitro: A Step Closer to 1-Stage Orthopaedic Procedures Click here for additional data file. Supplemental material, sj-pdf-1-ajs-10.1177_03635465211055164 for Device-Based Enrichment of Knee Joint Synovial Cells to Drive MSC Chondrogenesis Without Prior Culture Expansion In Vitro: A Step Closer to 1-Stage Orthopaedic Procedures by Ala Altaie, Thomas G. Baboolal, Owen Wall, Hemant Pandit, Elena Jones and Dennis McGonagle in The American Journal of Sports Medicine
