# Supplementary material for: Device-Based Enrichment of Knee Joint Synovial Cells to Drive MSC Chondrogenesis Without Prior Culture Expansion In Vitro: A Step Closer to 1-Stage Orthopaedic Procedures
Source: Am J Sports Med. 2021 Nov 15;50(1):152–61. doi: 10.1177/03635465211055164 (PMC8739599; doi:10.1177/03635465211055164)
Supplement: sj-pdf-1-ajs-10.1177_03635465211055164 – Supplemental material for Device-Based Enrichment of Knee Joint Synovial Cells to Drive MSC Chondrogenesis Without Prior Culture Expansion In Vitro: A Step Closer to 1-Stage Orthopaedic Procedures [file sj-pdf-1-ajs-10.1177_03635465211055164.pdf]

Device Based Enrichment of Knee Joint Synovial CellsTo Drive MSC Chondrogenesis without Prior Culture Expansion in vitro - A Step Closer to One Stage Orthopaedic Procedures

Appendix

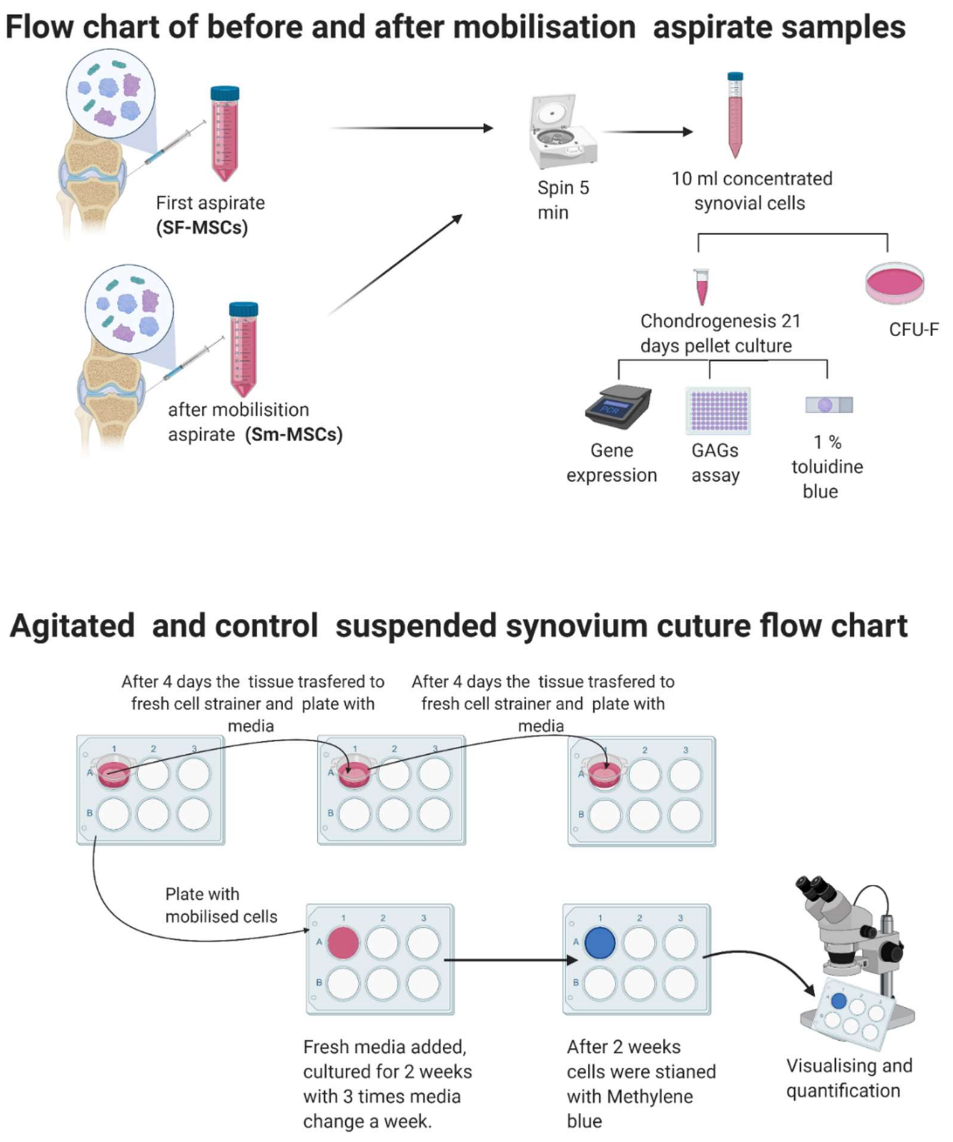

Figure A1: Experimental design and sample distribution for before after use of STEM device; 2ml from the 10 ml concentrated synovial cells were subjected to CFU-F for each petri dish and 2 ml for each chondrogenic pellet. And Experimental design for **synovium suspended culture. This figure created with BioRender.com**

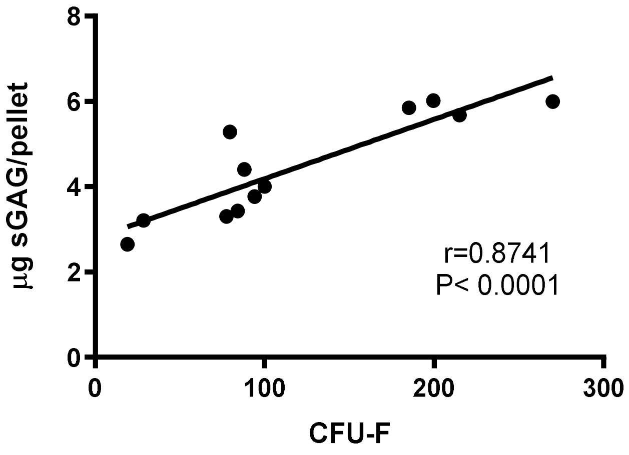

Figure A2:

Correlation of CFU-F numbers and sGAG production for SF-MSCs and Sm-MSCs

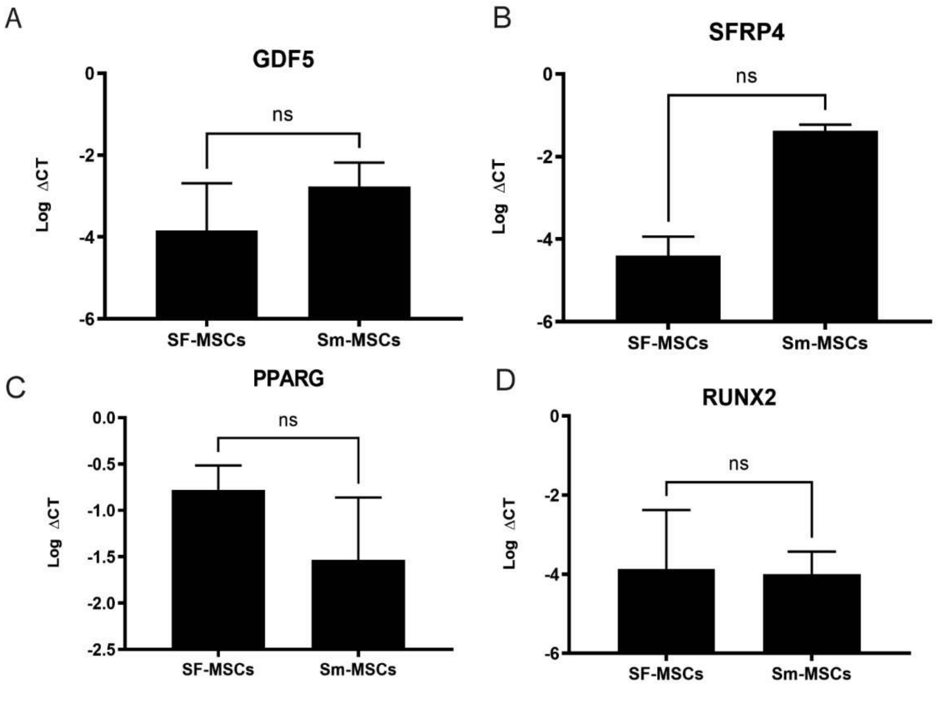

Figure A3: Gene expression of minimally manipulated SF-MSCs and Sm-MSCs chondrogenic pellets. A-B synovial origin MSCs marker. C: Adipogenic marker. D: Osteogenic marker.

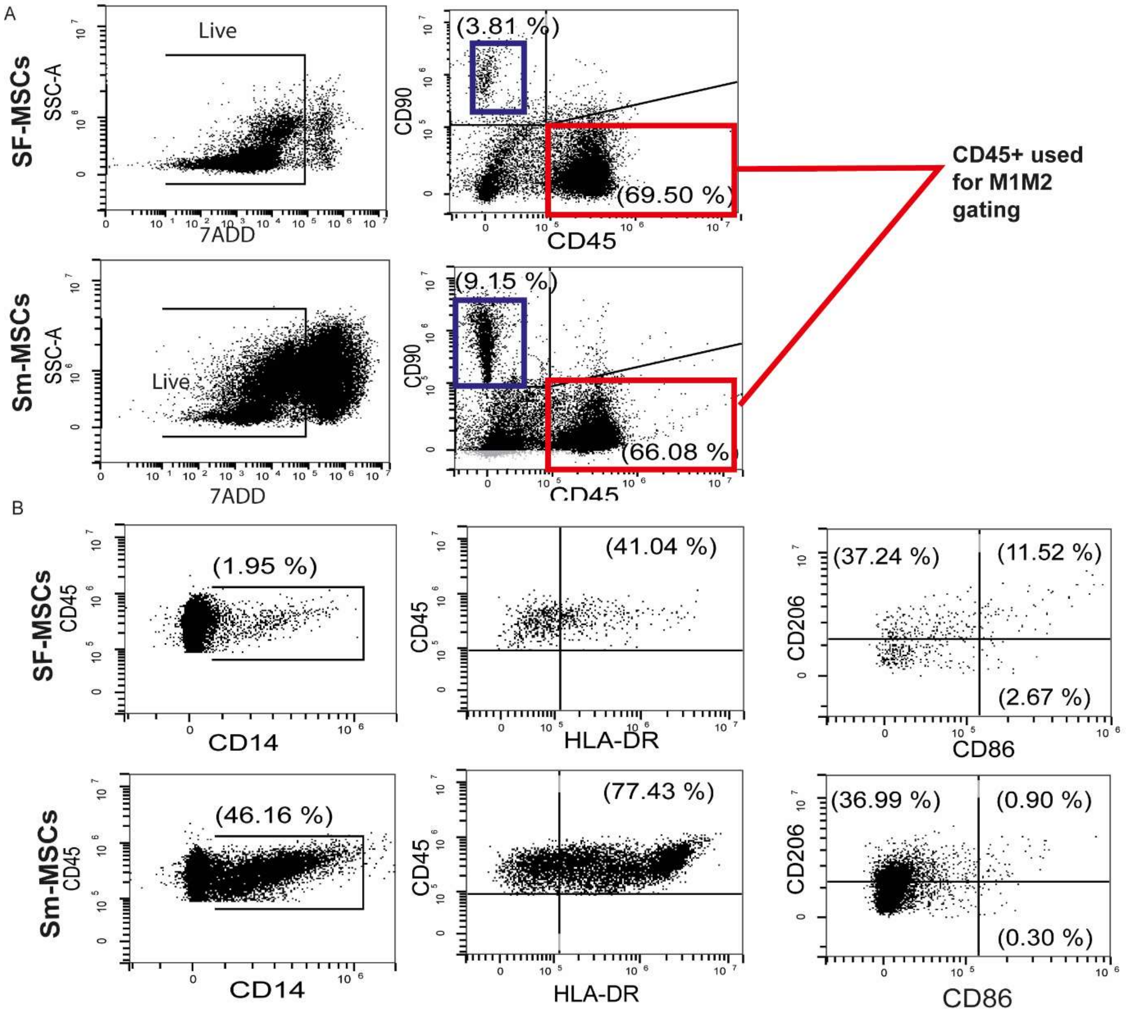

Figure A4: Gating strategies of CD90^{High}CD45^{Low} and macrophages subpopulations in non-expanded initial aspiration and mobilized joint aspirates (n=6).

Supplementary Methods

Retrieval of MSCs from irrigation fluid and synovial MSCs mobilization aspirating:

The first sample contained resident SF-MSCs and was collected by the initial saline used to irrigate the joint cavity. The second sample of irrigation fluid collected after agitation of the synovium to mobilize MSCs (Sm-MSCs) as previously described.[1] Briefly, the synovial membrane was brushed using a device to that biophysically augments release of stem cells into the synovial fluid.

The first sample was containing SF-MSCs was procured following injection of normal saline irrigation fluid as previously described[1] whereby approximately 45mLs of fluid on average could be retrieved. The second sample was collected after agitation of the bristled surface of the synovial surface for 1 minute. synovium using the stem cell mobilizing device (STEM device) releasing material containing synovial membrane MSCs (Sm-MSCs) about 43ml on average was retrieved, as previously described.[1]

Colony-forming unit–fibroblast (CFU-F) assay

For the CFU-F assay 2mL of the freshly obtained SF cells (from the concentrate 10 mL total volume) were plated in duplicate 10cm^2 plastic Petri dishes (1mL each) containing 15mL of StemMACS MSCs expansion media supplemented 100units/mL penicillin and 100 mg/mL streptomycin (all from Gibco). Cells were incubated in a humidified atmosphere at 37°C and 5% CO_2, with a full media change after 48 hours, followed by half media changes three times a week. Colonies were stained with 1 % methylene blue after two weeks of culture for counting using ImageJ version 2.0.

[1] Baboolal TG, Khalil-Khan A, Theodorides AA, et al. A novel arthroscopic technique for intraoperative mobilization of synovial mesenchymal stem cells. *Am J Sports Med.* 2018;46(14):3532-3540.
